# Healing After Horizontal Root Fracture of Maxillary Central Incisor: A Case Report With 24-Month Follow-Up

**DOI:** 10.7759/cureus.43373

**Published:** 2023-08-12

**Authors:** Noopur A Mane, Pradeep Shetty, Anamika C Borkar, Sanchit V Mujumdar, Aadia Mujawar

**Affiliations:** 1 Department of Conservative Dentistry and Endodontics, Dr. D. Y. Patil Dental College and Hospital, Dr. D. Y. Patil Vidyapeeth, Pune, IND; 2 Department of Conservative Dentistry and Endodontics, Dr. D. Y. Patil Dental School, Pune, IND; 3 Health Administration, Cornell University, Ithaca, USA

**Keywords:** emergency endodontic treatment, maxillary incisors, healing, trauma, horizontal root fracture

## Abstract

Facial traumatic injuries are quite common, resulting in the fracture and displacement of tooth and/or bone fragments. Loss of tooth structure may have lifetime consequences for the patient. Horizontal root fractures (HRFs) may occur in the maxillary anterior teeth at different locations, showing varied patterns of healing. The treatment options range from simple observation and follow-ups to conservative management or even complex surgical procedures. Correct and timely intervention can help preserve the tooth structure, leading to the long-term survival of the tooth. The present case report showcases endodontic treatment and favourable healing of a horizontal root fracture at the apical third of a permanent maxillary central incisor. At the 24-month follow-up, the tooth is clinically healthy, and radiographic images show a successful outcome.

## Introduction

Trauma to the dentoalveolar region is very common, more so in children and teenagers [1]. It may result in the fracture and displacement of tooth and/or bone fragments. Injuries to the soft tissues may also occur, which include contusions, abrasions, and/or lacerations. Loss of tooth structure may result in lifetime consequences for the patient [2].

In permanent dentition, the occurrence of horizontal root fractures (HRF) is less frequent, ranging from 0.5% to 7% of all dental trauma cases [3]. HRF occurs commonly in the anterior maxillary teeth, usually due to a frontal impact [4]. A classification of HRFs has been provided in the literature according to the location of the fracture line: apical, middle, or coronal third [5].

The prognosis depends on the patient’s age, the status of root development, displacement of the crown, location, and orientation of the fracture. Mobility, if present in the coronal portion, needs to be evaluated [6]. The more apical the root fracture is located along the length of the root, the better the prognosis. Management of the same can be done by root canal treatment (RCT) involving only the coronal fragment, RCT involving both fragments up to the root apex, or RCT of the coronal fragment followed by apicectomy of the apical fragment. Along with the treatment, the healing of the fracture needs to be assessed periodically. The pulpal tissues and PDL complex compete to repair the fracture line. When managed properly using the clinical guidelines, root fractures have shown a 10-year survival rate of 87% [7].

The present case report details a 24-month follow-up of a horizontal root fracture showing favourable healing with the closure of the inter-fragmentary space.

## Case presentation

History and diagnosis

This case report follows the 'PRICE 2020 Guidelines for Reporting Case Reports in Endodontics'. [8]

**Figure 1:**
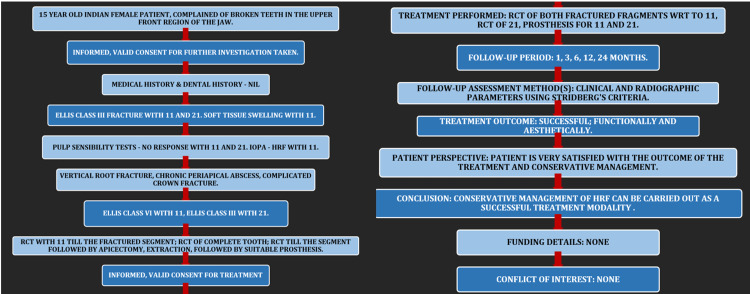
PRICE 2020 guidelines depicting the steps involved in reporting this case report. IOPA: intra-oral periapical radiograph, HRF: horizontal root fracture, RCT: root canal treatment.

A 15-year-old female patient reported to the hospital with a chief complaint of broken teeth in the upper front region of the jaw. The patient gave a history of falls two to three months prior to reporting to the institution. The trauma resulted in a fractured maxillary right central incisor (11) and maxillary left central incisor (21). The medical and family history of the patient were non-contributory. On intraoral examination, soft tissue swelling was seen clinically on the labial mucosa at the apical region of 11, as seen in Figure 1A.

No sinus tract, gingival recession, pockets, or mobility were noted. Tooth 11 and 21 showed no response on electric pulp testing or the cold test. On radiographic examination, an intra-oral periapical radiograph (Figure 1B) showed oblique fracture lines involving the enamel, dentin, and pulp extending from the middle to the incisal third of the crowns of 11 and 21. An HRF was seen at the junction of the middle and apical thirds of 11. Periapical widening and loss of lamina dura were noted at the apical third of 11. From the above findings, a fracture of the root with loss of crown structure, or Ellis class VI fracture, was diagnosed with 11, and a coronal fracture exposing the pulp, or Ellis class III fracture, was diagnosed with 21.

As the pulp was necrosed and infected, it was decided to treat both the coronal and apical root fragments of tooth 11, followed by the RCT of tooth 21. Written informed consent was taken from the patient’s guardian after explaining the procedure and prognosis of the same.

Clinical procedure and follow-up

**Figure 2:**
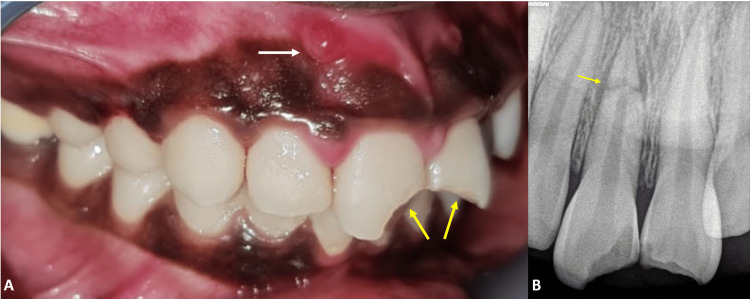
(A) Pre-operative clinical image showing soft tissue swelling at the apical region of 11 as represented by the white arrow, fracture lines at the coronal portion of 11 and 21 as represented by the yellow arrows. (B) Pre-operative radiograph with the yellow arrow showing HRF at the junction of the middle and apical third of tooth 11. HRF: horizontal root fracture.

Local anaesthesia solution, 2% lignocaine hydrochloride with 1:200,000 adrenaline (Xicaine, ICPA Health, India), was administered, and after rubber dam isolation, RCT was initiated (Figure 2A). A round carbide bur was used for the access opening. Working length was measured after negotiating a 10K (Mani, Inc., Shioya, Japan) file through the fracture line using an electronic apex locator (Root ZX, J Morita Corp., Tokyo, Japan), and confirmed on the radiograph. Chemo-mechanical tooth preparation was done up to size F2 (Protaper Universal, Dentsply Maillefer, Ballaigues, Switzerland) with hand files. Intermittent irrigation was done using 3% sodium hypochlorite (NaOCl) (Prime Dental Products Pvt. Ltd., India) and normal saline. A chelating agent, 17% ethylenediaminetetraacetic acid (EDTA) (RC Help, Prime Dental Products Pvt. Ltd., India), was used between each file change. A calcium hydroxide dressing (RC Cal) was placed in the canal. A temporary restoration was given (Cavit G, 3M ESPE), and systemic antibiotics (amoxicillin/clavulanic acid 500 mg + 125 mg every eight hours for seven days) were prescribed. The patient was recalled after two weeks. At the second appointment, the swelling had subsided. A triple antibiotic paste (TAP) - a freshly made mixture of ciprofloxacin, metronidazole, and minocycline (1:3:3) - was delivered into the canal with a lentulospiral, 3 mm short of working length [9]. On the final visit two weeks later, TAP was flushed out using normal saline. The final irrigation was done with 2% chlorhexidine (Dentachlor). As an adjunct, passive ultrasonic irrigation was also performed with an ultrasonic tip inserted into the canal. (Ultra X, Orikam). Obturation was done using the lateral compaction technique. The master cone (25/0.06) was used along with accessory cones (15/0.02), and the canal was obturated at the apex, through the fracture line. A calcium hydroxide-based sealer (Sealapex, Kerr Dental, Brea, CA) was used. The obturation material through the fractured segments mimicked an intra-radicular splint. RCT for 21 was also initiated and completed in subsequent visits in a similar way. Post-obturation restoration for both teeth was done using bulk-fill composite resin added incrementally (GC Solare Sculpt, Tokyo, Japan) after one week, as seen in Figure 2B. The patient was kept on regular follow-ups for 1, 3, 6, 12, and 24 months. Clinically, the teeth were assessed for tenderness, mobility, depressibility, status of occlusion, presence of the sinus tract, and overall oral hygiene status. Radiographic evaluation was done for the presence of cracks, continuity of the lamina dura, bone loss, and radiographic changes indicating external or internal root resorption. Healing was evaluated using Strindberg’s criteria [10]. At the end of 12 months, the patient was asymptomatic, and hence, porcelain fused to metal crowns were fabricated for both 11 and 21.

**Figure 3:**
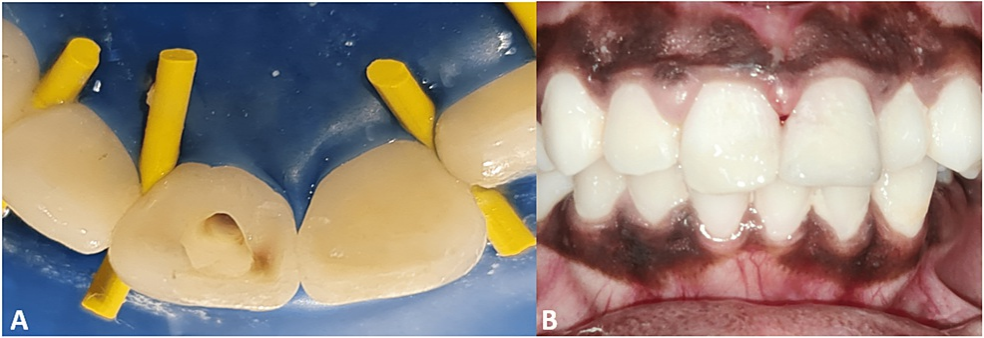
(A) Access cavity preparation after placement of rubber dam; (B) post-obturation restoration.

Radiographs were taken at each step and recorded (Figures 3A-3F).

**Figure 4:**
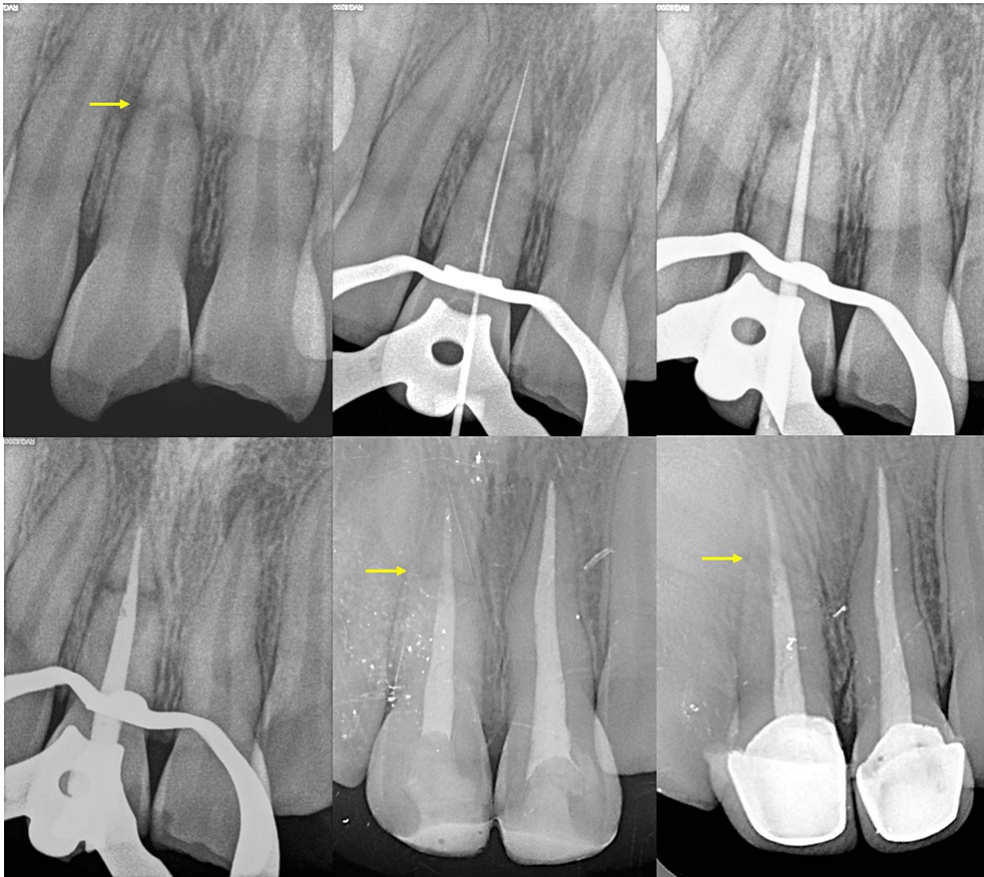
(A) Pre-operative radiograph showing HRF in 11 as represented by the yellow arrow; (B) working length determination; (C) master cone selection; (D) obturation of both fragments till the apex; (E) post-obturation restoration and 12 months follow-up. Yellow arrow shows healing of the inter-fragmentary space. (F) Placement of prosthesis and 24 month follow-up. Yellow arrow shows complete healing and hard tissue repair.

At the end of 24 months, no tenderness, mobility, depressibility, sinus tract, or swelling were seen. The same overjet and overbite were noted as before. Radiographically, hard tissue healing is observed in the interfragmentary space (Figure 3E-3F). Intact PDL space and lamina dura are seen. There is no periapical radiolucency or cracks, and there are no signs of bone loss or root resorption (Figure 4).

## Discussion

Root fractures often go undetected during the early management of traumatic injuries. This is because they can only be viewed after proper radiographic investigations or scans [7]. HRF requires dentists to have an in-depth understanding and knowledge of etiopathogenesis, tissue responses, treatment protocols, and prognosis.

The healing pattern is often complex as the dentin, pulp, periodontal ligament (PDL), and cementum undergo injuries [5]. When there is trauma to the tooth by a hard/blunt object, the impaction forces are transmitted to the root due to the increase in the area of resistance to the force received by the crown. This results in a root fracture, which usually occurs in the coronal or cervical portion of the root. Most pulps survive the injury. They remain healthy and function normally. Hence, root canal treatment is not usually indicated. If required, only the coronal root fragment needs to be treated, as the pulp in the apical fragment is usually unaffected, does not undergo necrosis, and remains healthy. However, some teeth may develop pulpal pathologies in the apical fragment, like irreversible pulpitis or an infected root canal system. In such scenarios, the treatment options are to either perform RCT of both the coronal and apical fragments or RCT of the coronal fragment followed by removal of the apical fragment (apicectomy) using a surgical approach [6-7]. Similar treatment modalities were shown by Sisodia and Manjunath for conservative and successful management of root fractures at different levels [11]. In the present case, the associated apical swelling indicated a diseased state of the pulp, and hence obturation was done of both fragments, considering the age and financial background of the patient. A calcium hydroxide dressing given prior to the obturation promotes the formation of a hard tissue barrier in the space between the two fragments. This also helps in confining the root-filling material within the canal space. This, along with the antibacterial action of calcium hydroxide, allows for internal hard tissue repair and a favourable prognosis [12-13]. It has been reported previously that TAP, when used for the disinfection of infected root dentin inside the canals, eliminates the bacterial flora in vivo [9,14].

Twelve months of regular follow-ups were done, where periapical radiographs were taken to check the adequate healing of the fragments and PDL formation. Percussion, palpation, and mobility tests were carried out [7]. Composite restorations have a limited lifespan and a tendency to fracture over time. This may further open pathways for bacteria to reinfect the canal. Hence, porcelain fused to metal (PFM) crowns were fabricated for both teeth after the composite restoration. This provided an additional seal and ensured no pathway for bacteria to enter and reinfect the root canal system in the future. As stated by Abbott in 2019, all teeth with root fractures that have been treated should be followed up in the usual manner: first, healing of the tissues in and adjacent to the fracture line should be ensured. A secondary assessment should be done to check that the tooth remains stable and that no reinfection of the root canal occurs. This can be assessed by radiographic observation of a reduction in the radiolucency and normal formation of bone and periodontal ligament [7,10,15].

The outcomes of root fractures could be of five types: (i) healing by hard tissue formation; (ii) healing by connective tissue formation; (iii) healing with interposition of bone and connective tissue; (iv) no healing but formation of granulation tissue; and (v) late pulpal necrosis followed by infection of the coronal fragment [7,15].

In the present scenario, the 24-month follow-up shows the closure of the space between the fractured segments. This could be due to connective tissue formation, where PDL cells are the dominant contributors. Re-modelling via resorption at the edges of the fracture line is a common phenomenon, creating rounded corners. Cementum formation may have also occurred, which grows into the space, reuniting the two fragments to some extent [5]. Regular follow-ups are necessary every four to five years to ensure the success of the treatment [7].

## Conclusions

Root fractures located at the apical third of the root have a good long-term prognosis. Conservative management of root fractures should always be carried out to preserve the maximum integrity of the tooth. Detailed knowledge along with good clinical skills are needed to achieve the best functional outcome without compromising aesthetics. This, coupled with periodic clinical and radiographic follow-ups, is essential to checking for the stability of the teeth. The present case shows excellent long-term healing of the fractured segments, indicating the future survival of the tooth.
